# Research Progress in the Field of Microbial Mitigation of Drought Stress in Plants

**DOI:** 10.3389/fpls.2022.870626

**Published:** 2022-05-19

**Authors:** Shifa Shaffique, Muhamad Aaqil Khan, Muhamad Imran, Sang-Mo Kang, Yong-Sung Park, Shabir Hussain Wani, In-Jung Lee

**Affiliations:** ^1^Department of Applied Biosciences, Kyungpook National University, Daegu, South Korea; ^2^Mountain Research Center for Field Crops Khudwani, Sher-e-Kashmir University of Agriculture Sciences and Technology of Jammu, Srinagar, India

**Keywords:** microbes, mitigate, drought, stress, plants

## Abstract

Plants defend themselves against ecological stresses including drought. Therefore, they adopt various strategies to cope with stress, such as seepage and drought tolerance mechanisms, which allow plant development under drought conditions. There is evidence that microbes play a role in plant drought tolerance. In this study, we presented a review of the literature describing the initiation of drought tolerance mediated by plant inoculation with fungi, bacteria, viruses, and several bacterial elements, as well as the plant transduction pathways identified *via* archetypal functional or morphological annotations and contemporary “omics” technologies. Overall, microbial associations play a potential role in mediating plant protection responses to drought, which is an important factor for agricultural manufacturing systems that are affected by fluctuating climate.

## Introduction

Despite several advances in agricultural technology, drought is a major environmental stress for plants. Interventions with microbes can mitigate drought stress (Hossain et al., 2016; Li and Liu, 2016; Salehi-Lisar and Bakhshayeshan-Agdam, 2016; Shinozaki et al., 2016). In this review, we summarized drought stress mitigation using microbes, current control tactics, and unconventional control approaches. We also discussed possible alternative strategies for more effective drought-stress management.

Drylands are used to evaluate drought stress in biomes because of their limited agricultural productivity. Given the importance of water as a macromolecule for the physiological growth and development of plants, water scarcity and deprivation are phenomena that commonly cause drought stress (Siddique et al., 2000; Bartels and Phillips, 2010; Farooq et al., 2012; Askari-Khorasgani et al., 2021). This is a major environmental constraint that limits crop production; indeed, drought stress affects physiological, biochemical, and molecular levels. In response to such stress, plants enter avoidance, tolerance, or adaptation phases (Palmer, 1965; Svoboda et al., 2002; Farooq et al., 2009; Basu et al., 2016). Lack of water affects the water-plant relationship, arrests the plant growth, and causes a reduction in the leaf size, stem extension, and root proliferation. Arrested plant growth leads to reduced carbon dioxide assimilation, which subsequently damages membrane potential. This damage leads to the accumulation of reactive oxygen species (ROS), with free radical accumulation leading to oxidative stress and disturbances in adenosine triphosphate synthesis (Anjum et al., 2011; Van Loon, 2015). Drought stress can be reduced through breeding, mass screening, and exogenous phytohormone production (Siddiqui et al., 2022). Plants can autoregulate to minimize stress by producing phytohormones [e.g., abscisic acid (ABA) and gibberellins] and low-molecular-weight osmolytes (e.g., amino acids and polyols) and by modifying succulent leaves to reduce transpiration loss (Seki et al., 2007; Farooq et al., 2009; Rahdari and Hoseini, 2012).

**FIGURE 1:**
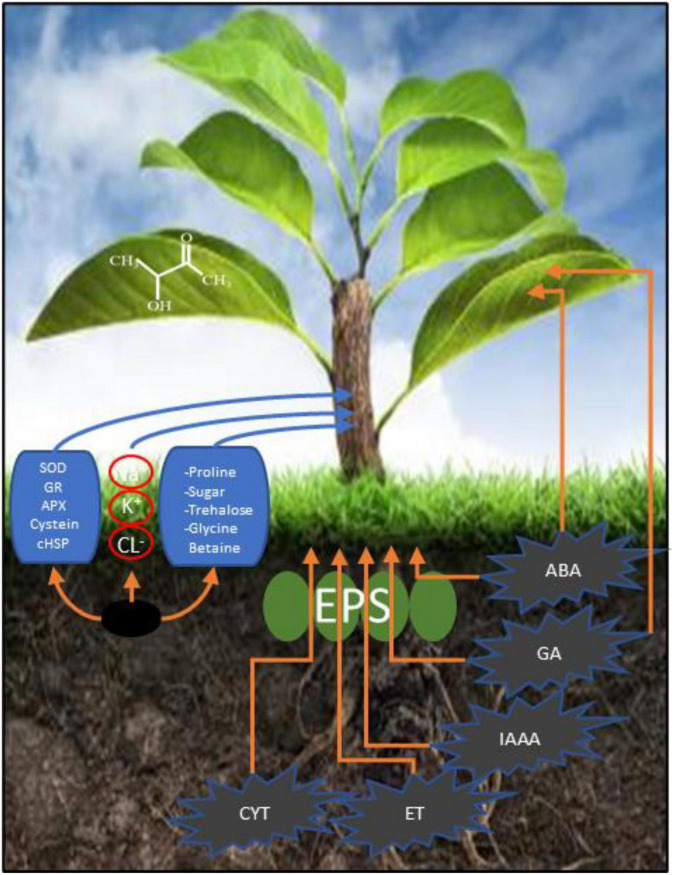
Mechanism of action of microbial mitigation of drought stress.

**FIGURE 2:**
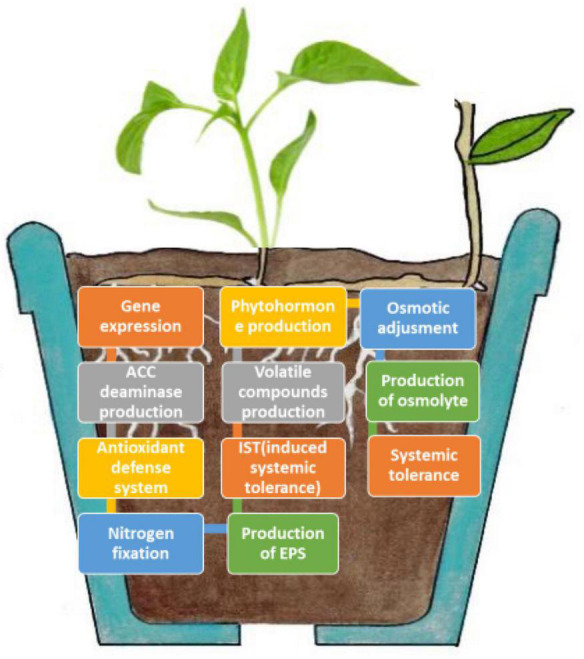
Plant–microbial interactions involved in the mitigation of drought stress.

**FIGURE 3:**
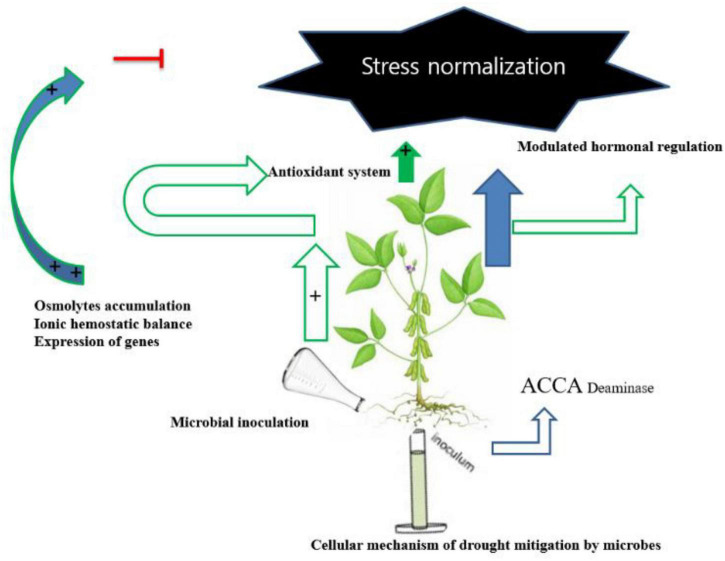
Cellular mechanism of drought mitigation by microbes.

Microbes can promote plant growth both directly and indirectly (Figure 1). The indirect activation of plant growth involves a series of events by which microbes prevent the inhibition of plant growth and development induced by pathogens (Ahmad et al., 2021). During direct activation, microbes biosynthesize bacterial compounds that promote the uptake of nutrients from the soil and stimulate plant growth and development (Berg, 2009; Choudhary et al., 2016). Microbes induce local or systemic stress mitigation response mechanisms that help plants survive under abiotic stress conditions (Figure 2), such as drought stress, and help plants sustain growth and development through the fixation, mobilization, and/or production of nutrients, hormones, and organic phytostimulants (Martins et al., 2018; Yadav and Yadav, 2018; Kour and Yadav, 2020). In this study, we summarized the currently known implications of using microbes for drought tolerance, including their fundamental mechanisms of action (Figure 3). This review adds to previously published reviews by addressing the implementation of mitigation strategies to prevent adverse effects of drought stress.

## Molecular Mechanism of Action of Microbes in Plants Under Drought Stress

### Microbial Production of Phytohormone

Phytohormones such as gibberellins, ethylene, indole acetic acid (IAA), ABA, and cytokinin are chemical messengers that are organic in nature, synchronize cellular events in plants, and play key roles in plant progression and drought stress (Kefeli et al., 2003; Khan et al., 2012; Jogawat et al., 2021).

Phytohormones are produced by plants and, in some cases, by microorganisms that interact with plants. Auxin mitigates drought stress in a secondary manner *via* cumulative root growth and modification of root design and/or root hairs, which absorb water and nutrients from the soil. Auxins are produced *via* tryptophan-dependent pathways (Vanneste and Friml, 2009; Zhao, 2010). They produce indole-3-acetamide, which is converted into indole-3-acetaldoxime and tryptamine to give the final product, i.e., indole-3-pyruvic acid (Skubacz et al., 2016). Several studies have provided evidence to support the role of IAA in inducing drought-related signaling pathways when plants are exposed to osmotic stress (Fahad et al., 2015; Yu et al., 2020). These adaptations are interrelated with higher auxin amalgamation in microbe-treated plants; auxin has the capacity to elongate the stems and coleoptiles of plants under stress conditions (Raheem et al., 2018; Ullah et al., 2019).

The application of microbes also enhances the production of plant gibberellins, which are diterpeniods that cause hyperactive elongation of stems in response to stress. Gibberellins are associated with two bioactive components, namely, carotenes and isoprene (Davies, 1995; Alagoz et al., 2018). Carotenes can stimulate photosynthesis and protect plant cells from harmful photodynamic reactions, whereas isoprene maintains turgor pressure and stabilizes the cell membrane structure (Jeon et al., 2003; Mollah and Pratiwi, 2020).

In response to abiotic stress, cytokinin biosynthesis increases in plants in association with auxin and regulation of developmental responses. Cytokinin can transfer the phosphoryl group P^+^O_3_^2–^. In addition, cytokinin helps in the phosphorylation of sugar molecules, which leads to their accumulation in cells and prevents reverse diffusion (Mok and Mok, 2001). Competitive phosphorylation helps plant cells combat stress, and cytokinin protects plant cells from oxidative stress (Werner and Schmülling, 2009; Wybouw and De Rybel, 2019).

Ethylene is an important hormone that regulates not only plant growth but also senescence of growth attributes. It frequently interacts with other hormones. Ethylene is a second messenger hormone that regulates various developmental processes, ranging from germination to the plant life cycle (Kanellis et al., 2012; Wen, 2014).

Abscisic acid is a particularly important hormone that is secreted when plants are subjected to stress; thus, it is known as a universal stress hormone. ABA plays an important role in mitigating stress because it causes stomatal closure and is a powerful inhibitor of stomatal opening (Bright et al., 2006). ABA also regulates genes responsible for desiccation tolerance (Sakata et al., 2014).

The ability of these rhizobacteria to synthesize and produce phytohormones is the first key factor in mitigating stress through a complex signaling network in plants to respond to environmental conditions and maintain the plant inner environment through regulation of these hormones, antioxidants, and osmolytes to enhance stress tolerance.

### Microbial Production of Aminocyclopropane-1-Carboxylate Deaminase

Aminocyclopropane-1-carboxylic acid (ACC) is a precursor of ethylene, the production of which increases in plants under ecological stress. Numerous microorganisms have been reported to produce ACC deaminase, which reduces ACC, thereby lowering the increased levels of ethylene in host plants (Campbell, 1995; Houda et al., 2013). Under drought stress, plants enhance their ethylene production, which inhibits plant growth by affecting seed germination and root enlargement. Higher ACC levels are induced to combat severe drought stress (Dikarev et al., 2016; Ustiatik et al., 2021).

Beneficial microbes can produce ACC deaminase. ACC regulates plant growth and development by sequestering and cleaving plant-produced ACC and lowering the level of ethylene in plants.

### Osmotic Amendment

When plants are subjected to abiotic stress, a sudden loss of osmolytes occurs, which can shock the plant. However, microbial interactions lead to osmotic adjustments. Plant–microbe interactions are facilitated by secreted metabolites; plants upregulate the production of metabolites such as proline, sugars, glycine, organic acids, trehalose, betaine, potassium, calcium, and chloride ions as an adaptive response to drought stress. As one of the most important osmolytes, proline accumulates in plants under osmotic stress and facilitates the maintenance and osmotic adjustment of cellular components in plant cells to overcome stress. Some rhizosphere bacteria produce proline (Yamada et al., 2005; Mohammadkhani and Heidari, 2008; Dobra et al., 2010).

Glycine betaine is a secondary metabolite that helps plants maintain growth and development. Trehalose is a safe, non-reducing sugar that contains two glucose molecules and stores energy for use under stress conditions. Microbes can accelerate trehalose biosynthesis through the TPS/trehalose-6-phosphate phosphatases (TPS/TPP) pathway, maintain osmolytes concentrations, and stabilize turgor pressure in plant cells (Iordachescu and Imai, 2011; Kahraman et al., 2019). The application of microbes to plant cells increases the secretion of organic acids (e.g., oxalic acid, citric acid, and malic acid) and minerals (e.g., chlorine, sodium, and potassium), which are important for nutrient availability, metabolic reactions, and the maintenance of osmoregulation in plant cells (Kirst, 1996; Chen and Jiang, 2010).

The ability to regulate osmotic amendment of plant-growth-promoting rhizobacteria (PGPR) in plant cells is due to generating a low water gradient potential in the cytosol. They maintain the turgor pressure and osmotic adjustment and improve stress tolerance.

### Microbial Production of Exopolysaccharides for Drought Stress Mitigation

Exopolysaccharides are macromolecules composed of long-chain polymers of repeating sugar units such as glucose, galactose, and rhannose in different ratios (Ilyas et al., 2020; Bhagat et al., 2021). Exopolysaccharides form hydrophilic biofilms that offer protection against aridness during osmotic stress by enhancing the water-retaining potential of the soil and regulating the distribution of biological carbon sources. Microbes form sheaths to protect roots from dehydration and maintain the moisture content (Sandhya et al., 2009; Nadeem et al., 2021). Exopolysaccharides can be released into soil as slime ingredients comprising cation hydrogen bridges, van der Waals linkages, and anion adsorption interactions, which act as defensive capsules around the soil and improve the biological properties of the soil by increasing aggregation and macroporosity (Ilyas et al., 2020). Overall, exopolysaccharide production helps plants cope with abiotic stress (Naseem et al., 2018).

The vital interaction of the plant and microbes facilitates the production of a biofilm that allows microbes to attach with plant roots, provides a shield to the roots, and imparts a strong root adhering capability.

### Effects of Microbial Volatile Organic Compounds Against Osmotic Stress

The application of beneficial microbes produces volatile compounds that can increase growth, development, photosynthesis, iron uptake, and crop productivity, while reducing the incidence of plant diseases and death. For example, stress-induced volatile compounds such as 3-hydroxy-2-butanone (acetoin), 2,3-butanediol, and 2-pentylfuran play a role in plant growth and development (Seco et al., 2015; Ali et al., 2017). These volatile compounds are also insect repellents owing to their strong odor and help boost plant growth. Under stress conditions, these compounds cause stomatal closure and impart systemic stress resistance to plants, highlighting their role in plant growth and development under stress conditions (Liu and Zhang, 2015; Mohammadipanah and Zamanzadeh, 2019).

Plant–microbial interactions emit volatile oil compounds (VOCs), which mitigate stress tolerance through the expression of genes and scavenge free radicals.

### Microbial Induction of Antioxidant Machinery in Plants

Osmotic stress induces the production of ROS in plants, including superoxide, radicals, singlet oxygen, hydrogen peroxide (H_2_O_2_), superoxide anion radicals, and alkoxy radicals. ROS react with lipids, proteins, and DNA, thereby causing oxidative damage and affecting redox regulation in plants (Tambussi et al., 2000; Ahmad et al., 2014). To protect against oxidative damage during osmotic stress, plants induce antioxidant defense systems involving enzymatic and non-enzymatic pathways (Chai et al., 2005). Enzymatic antioxidant pathways involve enzymes such as glutathione reductase, superoxide dismutase (SOD), catalase, and ascorbate peroxidase, whereas non-enzymatic components include ABA, cysteine, and glutathione (Chai et al., 2005; Ahmad et al., 2014; Li B. et al., 2020). The presence and exogenous inoculation of microbes in the soil can confer drought tolerance by inducing antioxidant systems. Various microbes, such as *Actinomycetes*, fungi, and algae, contain phenolic components that are secreted when plants are subjected to stress (Kaushal and Wani, 2016; Ilyas et al., 2021).

The ROS scavenging ability of PGPR regulates the antioxidant enzymes and may provide a solid barrier against abiotic stress. The plant–microbial interaction ensures that there is always a homeostatic balance between ROS and removal machinery, which mitigates stress tolerance in plants.

### Stimulation of Stress-Response Genes by Plant–Microbe Interactions

Stress tolerance can be enhanced by treating plants with microbes because this induces stress-response genes that regulate plant stress. The expression of such genes is modulated in plants under drought stress, which helps optimize plant growth and development. Stress-related genes and proteins involved in plant–microbe interactions include *CaPR-10*, *sHSP*, Δ1-pyrroline-5-carboxylate reductase (*P5CR*), dehydrin-like protein (*Cadhn*), vacuolar ATPase (*VA*), and pyrroline-5-carboxylate dehydrogenase (*P5CDH*) (Shinozaki and Yamaguchi-Shinozaki, 1998, 2007). Microarray studies have suggested that gene expression can be categorized into functional or regulatory proteins depending on the role of the encoded protein. Regulatory proteins include transcription factors, protein phosphate kinases, and ABA biosynthetic factors. Functional proteins include detoxification enzymes, water channel transporters, proteases, osmolyte biosynthesis enzymes, and macromolecule protection factors. Genes and transcription factors can encode sustainable agricultural products (Shinozaki and Yamaguchi-Shinozaki, 1998, 2007; Kavar et al., 2008).

The expression and upregulation of stress-related genes in response to drought stress can be used as a powerful tool for mitigating and enhancing drought tolerance in plants.

### Microbes and Mineral Uptake

Drought stress causes an imbalance between the mineral and ion exchange. It disturbs the levels of Na^+^, Cl*^–^*, and K^+^, which are responsible for plant growth. Plant–microbial interactions are able to maintain the ionic balance by scavenging the NHXI protein in the plant cytosol (Mukerji et al., 2006; Banfield and Nealson, 2018).

Beneficial microbial populations produce large numbers of organic components that can be used as signaling molecules and nutritional components for plants under drought stress. Various microbiota, such as fungi, cyanobacteria, and plant growth-promoting rhizobacteria, play important roles in mineral uptake and rehabilitation of the nutritional status (Teotia et al., 2016; Zhu et al., 2016). They mediate the solubilization of phosphosulfate compounds, nitrogen fixation, denitrification, and siderophore production. There are two pathways for mineral uptake, namely, direct and indirect (Zhu et al., 2016; Kapadia et al., 2021). Phosphate is present in the ecosystem mostly in the form of inositol phosphate; therefore, microbes solubilize phosphorus into the most soluble form by synthesizing low-molecular-weight components such as citric acid and gluconic acid. This is an example of an indirect pathway: in direct pathways, phosphorus is solubilized by lowering the pH of the external environment and turns into low-molecular-weight organic compounds (Hupfauf et al., 2016; Sharma et al., 2020).

Therefore, a microbial population that interacts with plants in a beneficial environment can provide hemostasis for ionic exchange in the cytosol by maintaining the nutritional status of plants under drought stress.

## Microbes That Mitigate Drought Stress

### Endophytes

**FIGURE 4:**
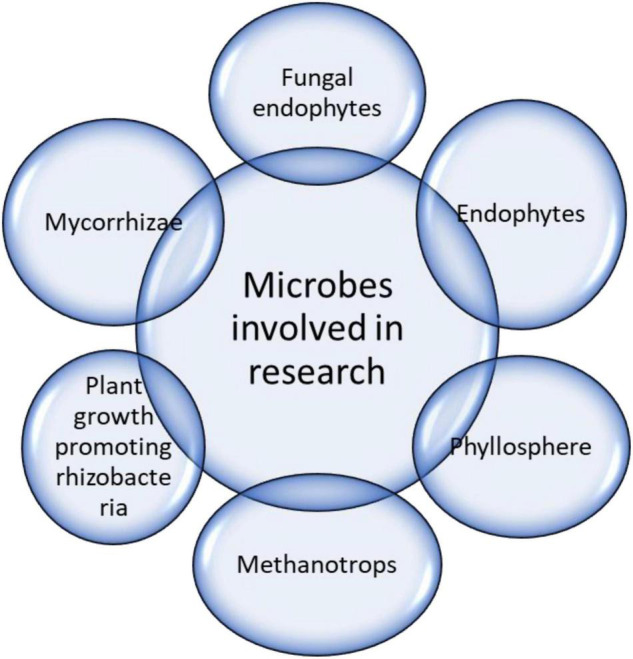
Microbes involved in drought stress mitigation.

Endophytes are microscopic organisms (i.e., bacteria, fungi, and viruses) that exhibit symbiotic relationships with plants (Figure 4). They have been comprehensively investigated because of their beneficial effects on environmental stress (Cheng et al., 2021; Meena et al., 2021; Verma et al., 2021). Endophytes can amplify biomass accumulation (fresh and dry) in host plants under stressful conditions (Zhang et al., 2006; Barman et al., 2016). The stress mitigation effects of the endophytes were comparable among different plant species. Under drought conditions in the presence of endophytes, eudicots and C4 plant species showed increased biomass compared with monocots and C3 species. Evidence of endophyte-mediated plant stress attenuation has been established in recent decades (Strobel, 2003; Rodriguez et al., 2009).

Microbes are beneficial for reducing stress, which would otherwise limit the crop yield. When plants are under stress, they experience difficulty in sustaining their normal growth. In a previous study (Singh et al., 2020), rice crops were subjected to drought stress, and various characteristics, such as growth limitation and metabolite content, were recorded. These plants were inoculated with a combination of *Trichoderma T42* and *Pseudomonas*, which significantly improved their metabolic ability during drought stress. Microbial induction increases the total polyphenolic content and activates antioxidant enzymes to reduce oxidative stress. Inoculation also led to overexpression of *PAL*, a gene responsible for combating drought stress. These results were significant compared with those of uninoculated plants (Singh et al., 2020).

In a comparative analysis, two groups were experimentally compared, namely, untreated red rice seedlings and red rice seedlings inoculated with *Gluconacetobacter diazotrophicus* strain Pal5 (Filgueiras et al., 2020). Both seedling types were grown under water constraints of 30–35, 50–55, 70–75, or 100% field capacity for 15 days. Physical, chemical, and molecular evaluations of the plants were subsequently conducted. Leaves were found to contain increased levels of osmoprotectants, such as osmolytes (e.g., proline and glycine betaine), which help combat drought stress. Omics analysis showed the relative gene expression of *gor*, *cat*, *P5CR*, *sod*, and *BADH*. Overall, this study suggests that *G. diazotrophicus* is effective at combating the effects of drought stress.

Currently, water scarcity is a major challenge; however, rhizobacteria can facilitate plant growth under water stress. A study conducted in India involved the inoculation of great millet plants with strains of *Streptomyces laurentii* (EU-LWT3-69) and *Penicillium* sp. (EU-DSF-10), which was followed by exposure to drought stress (Kour et al., 2020a). Under drought stress, plants experience various issues, including reduced bioavailability of phosphorus, which is an important component of the soil present in its active form in microbes that are precursors to the initiation of the phosphorus availability cycle. Both the tested bacterial strains, namely, EU-DSF-10 and EU-LWT3-69, tended to solubilize phosphorus, which increased its availability to plants. These two bacterial strains are also known to solubilize phosphorus. Phosphorus-solubilizing microbes also increased the chlorophyll content, proline levels, and glycine betaine levels while reducing lipid peroxidation. Overall, the results of this study suggest that inoculated plants show better growth and defense under drought conditions than those of uninoculated plants (Kour et al., 2020a). In a study (Bilal et al., 2020), the combined effects of LHL10 and LHL06 inoculation on *Glycine max* were investigated. The microbes produced a significant synergistic effect, with observable increases in the dry biomass, roots, shoot length, and leaf area. The genomic analysis revealed an increase in *HSP90* expression levels, while the biochemical analysis revealed that lipid peroxidation increased by 24.8–141.6%. Additionally, increased calcium levels and phosphate-solubilizing potential were observed. In conclusion, the synergistic effects were remarkably effective against combined stresses, as shown in Table 1 (Bilal et al., 2020).

**TABLE 1:** Endophytic microbes mitigate drought stress.

Country, year, references	Microbes	Host plant	Effect on drought stress	Mechanism of action	Isolation
India, 2020, (Singh et al., 2020)	*Trichoderma* T42 and *Pseudomonas*	*Oryza sativa* L.	Reduce	*PAL* expression, polyphenols	GenBank Accession No. JN128894; Banaras Hindu University, Varanasi, India
India, 2020, (Kour et al., 2020c)	EU- LRNA-72 and EU*-*FTF-6	Foxtail millet	Mitigate	↑Glycine betaine, chlorophyll a and b, proline, and sugars ↓LPO	Rhizosphere soil, Himachal Pradesh, India
Brazil, 2019, (Filgueiras et al., 2020)	*Gluconacetobacter diazotrophicus* Pal5	*Oryza sativa* L.	Defense	Expression of *gor*, *cat*, *P5CR*, *sod*, and *BADH* ↑proline ↑glycine betaine	Sugarcane, sweet potato
India, 2020, (Kour et al., 2020a)	*Streptomyces laurentii EU-*LWT3-69 and *Penicillium sp.* strain EU-DSF-10	*Sorghum bicolor* L.	Adaptation	↑osmolytes ↑ACC and deaminase ↑solubilize P ↓LPO ↑chlorophyll content	Rhizosphere, hamaliyah soil
Chile, 2019, (Silambarasan et al., 2019)	CAM4	*Lactuca sativa*	Mitigate	↓MDA ↑proline ↑chlorophyll a and b and carotenoids	Rhizosphere soil, Araucania region, Southern Chile
South Korea, 2019, (Bilal et al., 2020)	Endophytes (LHL10 and LHL06)	*Glycine max*	Drought protection	↑*GmHsp90A2* and *GmHsp90A1* expression ↑SOD ↓LPO	Endophytic microbes
Brazil, 2018, (Martins et al., 2018)	UFGS1, UFGS2, UFGRB2, and UFGRB3	*Glycine max*	Reduce	Gmdreb1a ↑stomatal conductance ↑transpiration ↑Fv/Fm	Rhizosphere, Goiania, Goiás, Brazil

*The ↑ and ↓ represent increase and decrease the response of specific mechanism.*

In another study conducted by Kang et al. (2021), two *Enterobacter ludwigii* strains, namely, AFFR02 and Mj1212, were isolated and inoculated into alfalfa plants; subsequently, their growth attributes, hormones, and mineral concentrations were assessed. The data showed that bacterial inoculation significantly rescued plants from drought stress. The stalk diameter, fresh and dry biomass, and root/shoot elongation were all significantly higher in the treated plants than in the untreated plants. In addition, treated plants have higher levels of ABA, flavonoids, and minerals than those of untreated plants (Kang et al., 2021).

Silambarasan et al. (2019) mixed the *Rhodotorula mucilaginosa* strain CAM4 with sawdust at a ratio of 1–5%, and the formulation was applied to *Lactuca sativa*. The inoculated microbial formulations displayed tolerance to drought stress at various developmental stages. An increase in the content of chlorophyll (48–65%), carotenoids (12–54%), and proline was observed. Proline is an important precursor for maintaining the cell structure and clearing oxidative stress. Plant growth and development were observed for 24 weeks, and growth, dry biomass, root proliferation, and stem elongation were all significantly improved in the treated group compared with those in the untreated group. Furthermore, malondialdehyde (MDA) levels decreased, indicating lipid peroxidation. Overall, the formulation was suitable for reducing aluminum, drought, and salinity stress and reduced oxidative stress by activating antioxidant enzymes (Silambarasan et al., 2019).

### Fungal Endophytes

Desert plants use fungal endophytes to mitigate salinity and drought stresses in arid environments. In a study conducted by Jain et al. (2020), tomato and cucumber seedlings were exposed to fungal endophytes for 96 days. These include halotolerant microbes, such as *Neocamarosporium chichastianum, Neocamarosporium goegapense*, and *Periconia macrospinosa*, which mitigate stress by increasing the levels of proline, antioxidant enzymes, and chlorophyll, as well as enhancing growth attributes. Among these, *P. macrospinosa* significantly reduced drought and salinity stress. Thus, some endophytes can adapt to particular habitats and act as biofertilizers and ecofriendly protectors against stress (Jain et al., 2020).

When the bacterial strain YNA59 was inoculated into a broccoli plant (Kim et al., 2020), a remarkable increase in hormone levels (e.g., ABA, jasmonate, and salicylic acid) was observed. Inoculated plants also showed significant enhancement in antioxidant enzyme levels, including SOD, catalase, and ascorbate peroxidase levels (Kim et al., 2020). Another study conducted to determine the role of the fungal endophyte *Neotyphodium coenophialum* when inoculated into *Lolium arundinaceum* under drought stress revealed that the mechanism of drought tolerance was dependent on the osmotic balance and increased water uptake efficiency, which enhanced photosynthesis and altered gene expression. In addition, endophytes isolated from a *Nicotiana* plant and inoculated into *Nicotiana benthamiana* increased the ability of the plant to tolerate drought stress (Dastogeer et al., 2020; Table 2).

**TABLE 2:** Fungal endophytes mitigate drought tolerance.

Country, year, references	Microbes	Host plant	Effect on drought stress	Mechanism of action	Isolation
Korea, 2020, (Kim et al., 2020)	YNA59	Broccoli	Mitigate	↑ABA, ↑sugar content ↑protein, ↑chlorophyll content, ↑JA, and ↑SA	Daehaw-myeon, Gang won-do, Republic of Korea
Bangladesh, 2020, (Dastogeer et al., 2020)	*Neotyphodium coenophialum*	*Lolium arundinaceum* *Nicotiana benthamiana*	Drought tolerant	↑gaseous exchange ↑antioxidant enzyme Altered gene expression and osmotic balance	Endophytic fungi from ferns, grasses, mosses, and pteridophytes

*The ↑ and ↓ represent increase and decrease the response of specific mechanism.*

Under low-water conditions, endophytic microorganisms associated with plants have the potential to enhance resistance by maintaining water status, ion homeostasis, and nutrient uptake to induce oxidative stress and enhance stress tolerance.

### Phyllosphere

Phyllosphere microbes are present above the ground surface of plants. These bacterial strains can promote plant growth (Figure 4). In one study, rice plant seedlings were selected and subjected to drought stress, and the plants were inoculated with the phyllosphere bacterial strains PB50, PB46, and PB3 based on their significant plant-growth-promoting activities in PEG 6000 plants subjected to drought (Arun et al., 2020). Growth attributes, such as root and shoot length, leaf area, and stem extension, were measured, and the biochemical analysis of the plants revealed their remarkable potential to solubilize important nutrients, including potassium, phosphorus, and zinc. Nutrient availability enables plant hydration under drought stress. In addition, the biochemical analysis revealed an increase in the levels of exopolysaccharides, phytohormones, soluble sugars, chlorophyll, total protein, and sugars, which improved plant survival under conditions of water constraints (Arun et al., 2020). In another study (Devarajan et al., 2021), rice phyllosphere bacterial strains (e.g., *Bacillus endophyticus* PB3, *Bacillus altitudinis* PB46, and *Bacillus megaterium* PB50) were inoculated into *Oryza sativa* pots in a greenhouse experiment. Drought tolerance was moderately minimized in strain PB50, resulting in significant tolerance to drought. Additionally, biochemical and morphological traits were observed and compared; inoculated plants showed higher expression of *LEA*, *SNAC1*, *HSP70*, *RAB16B*, and *bZIP23* as well as osmolyte accumulation, which helped combat drought (Devarajan et al., 2021; Table 3).

**TABLE 3:** Phyllosphere mitigates drought stress.

Country, year, references	Microbes	Host plant	Effect on drought stress	Mechanism of action	Isolation
India, 2020, (Arun et al., 2020)	Phyllosphere bacteria (PB50, PB46, and PB3)	Rice plant *[R(4), PMK3]*	Tolerant	↑chlorophyll, carotenoids, proteins, soluble sugar, and exopolysaccharides	Leaf surface of drought-tolerant plant
India, 2021, (Devarajan et al., 2021)	Rice phyllosphere bacteria *(Bacillus endophyticus PB3, Bacillus altitudinis* PB46, and *Bacillus megaterium* PB50)	*Oryza sativa* L.	Moderate	Overexpression of genes (↑*ABF*, *OsbZIP23*, and *bZIP23*) Minimal oxidative damage Osmolyte accumulation	Rice phyllosphere

*The ↑ and ↓ represent increase and decrease the response of specific mechanism.*

These beneficial microorganisms reside on the ground surface of plants. Phyllosphere microbiota enhance drought tolerance by promoting plant growth and protecting plants.

### Plant-Growth-Promoting Rhizobacteria

Plant-growth-promoting rhizobacteria can ameliorate drought stress and improve agronomic sustainability (Kanwal et al., 2017; Vanamala et al., 2021; Aslam et al., 2022). PGPR alleviate drought stress *via* rhizobacteria-induced drought endurance and resilience (RIDER), which induces biochemical changes (Rubin et al., 2017; Khan et al., 2018; Ansari and Ahmad, 2019). Various RIDER mechanisms include optimization of the antioxidant defense system, bacterial exopolysaccharides, phytohormone production, and cyclic metabolic pathway conventions that are involved in the deposition of several carbon-based components (Figure 1), such as sugars, amino acids, and polyamines, as well as the production of heat-shock proteins (Figueiredo et al., 2010; Monteiro et al., 2021; Sinha et al., 2021).

Plant-growth-promoting rhizobacteria can effectively mitigate drought stress in wheat and *Zea mays* grass. In one study (Jochum et al., 2019), two microbial strains, namely, *Bacillus* sp. 12D6 and *Enterobacter* sp. 16i, were used to combat drought stress. Plant growth attributes, hormones, and metabolite contents were analyzed. The results suggest that *Bacillus* sp. 12D6 was more effective in mitigating drought stress by increasing the root length, surface area, and plant productivity. The phytohormones IAA and salicylic acid were also found to be partly responsible for drought stress tolerance (Jochum et al., 2019). Bano et al. (2013) inoculated *Azospirillum* (GQ255950) into *Z. mays* plants. Microbes were isolated from water-restricted environments and inoculated into plants during the vegetative phase. After inoculation, the plants were subjected to drought conditions, and their growth attributes were recorded. Inoculation increased the root length, fresh biomass, and shoot length by 2.69–9.70%. Inoculation also increased the levels of proline, soluble sugars (63.15%), amino acids (54.54%), and osmoticum. Overall, inoculated plants can cope better with drought stress than uninoculated plants (Bano et al., 2013).

Drought is an important biotic stress factor that negatively affects crop yield. In a previous study, wheat plants were inoculated with *Pseudomonas libanensis* EU-LWNA-33, and the measurement of growth attributes revealed that the root length and biomass increased following inoculation. Furthermore, the biochemical analysis showed that proline levels increased by up to twofold, whereas glycine betaine levels increased by up to 1.2-fold at 75% stress. Proline and glycine betaine levels affect osmoregulation, as well as the solubilization and uptake of phosphorus, which is an important macronutrient with limited availability in plants. The inoculated strains effectively solubilized phosphorus (Kour et al., 2020b).

Drought stress can alter microbial interactions; for example, in a greenhouse experiment, three types of soil obtained from California valley rice fields were subjected to drought stress (Santos-Medellín et al., 2017). Water restriction altered the microbial interactions in all soil types. The altered patterns comprised enriched *Actinobacteria* and *Chloroflexi* and loss of *Acidobacteria* and *Deltaproteobacteria*. Compartment-specific restructuring results in plant survival under drought conditions (Santos-Medellín et al., 2017).

Plant-growth-promoting bacteria (PGPB) can help mitigate drought stress in various ways. Phytohormone production, 1-aminocyclopropane-1-carboxylate deaminase exopolysaccharide activation, solute production, chlorophyll synthesis, and increased mineral solubilization can help reduce stress. Such bacteria are safe, eco-friendly, and help sustain drought-tolerant crops (Priyanka et al., 2019). In a previous study (Chiappero et al., 2019), PGPR were isolated from soil, inoculated on Lysogeny broth (LB) medium plates, and cultured for 24 h in an incubator. After 1 day, the plate media contained colonies of *Pseudomonas fluorescens* WCS417r and *Bacillus amyloliquefaciens* GB03, both of which were evaluated for their effects on drought stress. Peppermint was selected, drought stress was induced, and plants were inoculated with microbes. The results demonstrated that drought stress was significantly reduced by the upregulation of the antioxidant defense system. Bioassay results revealed the hallmarks of phenolic components (Chiappero et al., 2019). In addition, the roots of *Acacia arabica* plants have been shown to be enriched with rhizosphere bacteria.

Plant-growth-promoting rhizobacteria such as *Bacillus*, *Enterobacter*, *Moraxella*, and *Pseudomonas* have been isolated and inoculated into drought-stressed wheat plants. The analysis of physiological and biochemical markers in these plants revealed increased levels of auxin, which help plants mitigate stress and enhance drought tolerance, thereby overcoming drought-related crop loss. *Bacillus* species can significantly improve auxin production to 25.9 μg ml^–1^, which, in turn, improves field capacity by 10% and crop yield by 34% (Raheem et al., 2018). PGPR strains such as *Pseudomonas putida* (i.e., NBRIRA) and *B. amyloliquefaciens* (i.e., NBRISN13) have been isolated from alkaline soils in Uttar Pradesh, India (Kumar et al., 2016). These two strains were simultaneously inoculated into chickpea plants to observe their combined effects in *in vivo* greenhouse and *in vitro* experiments. Their combination in plants led to an increased chlorophyll content, photosynthesis, osmolyte content, and biomass compared with those in single-strain treatments. Thus, PGPB are ecofriendly biofertilizers that can alleviate drought stress (Kumar et al., 2016). Various studies on several microbes have provided a basis for studies on microbial interactions. These studies have investigated the dynamics of microbial interactions to reveal their combined effects on stress tolerance in plants. In a field study, *Azospirillum brasilense* Sp245 was inoculated into wheat seedlings, and comparisons were made between inoculated and uninoculated seedlings (Creus et al., 2004). The inoculated seeds contained a higher mineral content, with increased levels of Mg, K, and Ca, and showed improved water regulation. Overall, a 12.4% improvement in grain yield was observed after inoculation.

*Actinomycetes* are Gram-positive anaerobic unicellular microbes that mitigate drought stress. However, research on this topic is scarce. In a previous study involving *Actinomycetes*, *Streptomyces pactum* Act12 was inoculated into the wheat plant cultivar Xinong 979 (Li H. et al., 2020). This inoculation significantly increased the overexpression of *EXPA6*, *P5CS*, *EXPA2*, and *SnRK2* and increased the root length by 13.6%, shoot length by 10.3%, and fresh biomass by 21.3%. Chemical assays also revealed that the exposed seedlings showed stress reduction through an increase in the levels of sugars and antioxidant enzymes.

Water shortages can be a major challenge in agricultural systems because they negatively affect the quality and quantity of crops. An experiment was conducted on foxtail millet crops that were subjected to drought stress, followed by inoculation with *Acinetobacter calcoaceticus* EU-LRNA-72 and *Penicillium* sp. EU-FTF-6 (Kour et al., 2020c). Plant growth attributes were measured, which showed mitigation of drought stress and physiological growth due to inoculation. In this study, drought stress was mitigated by the accumulation of osmolytes, proline, and glycine betaine, as well as by increased levels of chlorophyll a and b. An increased chlorophyll content resulted in plant growth and development, whereas increased proline and glycine betaine levels improved osmotic adjustment and membrane integrity, respectively. These results demonstrated that inoculation with microbes efficiently combats drought stress (Kour et al., 2020c). In a randomized controlled trial (Martins et al., 2018), soybean was inoculated with *Bacillus subtilis* (i.e., UFGS1), *Bacillus thuringiensis* (i.e., UFGS2 and UFGRB2), and *Bacillus cereus* (i.e., UFGRB3) strains. The strains were selected based on their growth under water stress on media plates. *B. thuringiensis* UFGRB2 maintained the quantum efficiency of PSII; *B. thuringiensis* and *B. cereus* maintained the photosynthetic rates of the plants, while *B. thuringiensis* sustained the transpiration rate and stomatal conductance. These physiological features demonstrate the improved growth observed in inoculated plants compared with that of uninoculated plants. Additionally, the genomic analysis showed that overexpression of *Gmdreb1a* contributed to drought stress tolerance (Martins et al., 2018; Table 4).

**TABLE 4:** Plant-growth-promoting rhizobacteria and drought tolerance.

Country, year, references	Microbes	Host plant	Effect on drought stress	Mechanism of action	Isolation
India, 2020, (Kour et al., 2020c)	*Acinetobacter calcoaceticus* EU-LRNA-72 and EU-FTF-6	Foxtail millet	Mitigate	↑glycine betaine, chlorophyll a and b, proline, and sugars ↓LPO	Rhizosphere, Himachal Pradesh, India
Brazil, 2018, (Martins et al., 2018)	*Bacillus* strains UFGS1, UFGS2, UFGRB2, and UFGRB3	Soybean	Reduce	Gmdreb1a ↑stomatal conductance ↑transpiration ↑ Fv/Fm	Rhizosphere, Goiania, Goiás, Brazil
United States, 2019, (Jochum et al., 2019)	*Bacillus* sp. (12D6) and *Enterobacter* sp. (16i)	*Triticum aestivum* and *Zea mays*	Tolerate	↑IAA ↑SA	Rhizosphere El Paso, TX, United States
Pakistan, 2013, (Bano et al., 2013)	*Azospirillum* (GQ255950)	*Zea mays*	Mitigate	↑amino acids, proline, and sugar	Water-stressed condition
India, 2019, (Kour et al., 2020b)	*Pseudomonas libanensis* EU-LWNA-33	Wheat	Sustain	↑ACC deaminase ↑osmolytes ↑P solubilization	Baru Sahib Valley of Divine Peace
Unites States, 2017, (Santos-Medellín et al., 2017)	Compartment-specific restructuring	*Oryza sativa* and *Oryza glaberrima*	Survival	↑*Actinobacteria* ↑*Chloroflexi* ↓*Deltaproteobacteria* ↓*Acidobacteria*	California Central Valley fields
India, 2019, (Priyanka et al., 2019)	*Azospirillum*	Wheat (*Triticum aestivum*)	Tolerant	↑phytohormone ↑solute formation ↑exopolysaccharide production	Rhizosphere soil, India
	*Azospirillum brasilense*	*Phaseolus vulgaris*	↑ACC deaminase ↑overexpression of *Cadhn*, *VA*, *sHSP*, and *CaPR-10*		
	*Achromobacter piechaudii*	*Lycopersicon esculentum* and *Capsicum annuum*			
	*Azospirillum brasilense*	*Zea mays*			
	*Bacillus licheniformis K11*	*Capsicum annuum*			
	*Achromobacter piechaudii* ARV8	*Lycopersicon esculentum* and *Capsicum annuum*	↓ethylene ↑ACC deaminase ↑root and shoot length ↑chlorophyll synthesis ↓ethylene ↓ACC deaminase		
	*Enterobacter cancerogenus*	*Jatropha curcas*			
Argentina, 2019, (Chiappero et al., 2019)	PGPR (*Pseudomonas fluorescens* WCS417↑r and *Bacillus amyloliquefaciens* GB03)	*Mentha piperita*	Mitigate	↑phenolic compounds Antioxidant defense	Rhizosphere soil
Pakistan, 2017, (Raheem et al., 2018)	PGPR (*Bacillus, Enterobacter, Moraxella*, and *Pseudomonas*)	*Triticum aestivum* L.	Mitigate	↑auxin	Root system of *Acacia arabica*
Canada, 2004, (Creus et al., 2004)	*Azospirillum brasilense* Sp245	*Triticum aestivum*	Minimize	↑mineral content ↑elastic adjustment	N/A
China, 2020, (Li H. et al., 2020)	*Streptomyces pactum* Act12	Wheat	Resistance	Overexpression of *EXPA2*, *EXPA6*, *P5CS*, and *SnRK2* ↑root length, ↑shoot length, ↑sugar content, ↑MDA, and ↑ABA	Alpine meadow in the Sanjiangyuan region
India, 2016, (Kumar et al., 2016)	PGPR (*Pseudomonas putida* NBRIRA and *Bacillus amyloliquefaciens* NBRISN13)	Chickpea	Tolerate	↑chlorophyll ↑antioxidant enzymes ↑protein content	Alkaline soil of Uttar Pradesh, India

*The↑ and ↓ represent increase and decrease the response of specific mechanism.*

Plant-growth-promoting rhizobacteria enhance the growth, development, and disease resistance of plants through a wide range of mechanisms. These are the most common microbes that mitigate drought stress. They secrete compounds of valuable potential biostimulants and play a pivotal role in plant stress responses.

### Methanotrophs

To sustain biofertilization in India, an attempt was made to isolate and inoculate a methylotrophic bacterial community (e.g., *Methylarcula*, *Methyloferula*, *Methylobacterium*, *Methylotenera*, *Methylobacillus*, *Methylophilus*, *Methylocapsa*, *Methylocella*, *Methylohalomonas*, *Methylomonas*, *Methylopila*, *Methylosinus*, and *Methylovirgula*) (Kumar et al., 2019). Bacteria were isolated from the phyllospheres of cotton, maize, earthworms, and rhizosphere soil in Tamil Nadu, India. Methylotrophs increased the levels of auxin and cytokines, and the increased levels of phytohormones promoted germination and growth. In addition to increasing the ACC deaminase activity, these bacteria enhance nitrogen fixation and mineral solubilization. The novelty of this microbial community is that it is composed of type 1 and type 2 methanotrophs that have the potential to tolerate stress (Kumar et al., 2019). In another study (Danish et al., 2020), rhizosphere bacteria were collected from *Z. mays* and reinoculated into plants; data (submitted to National Center for Biotechnology Information (NCBI)) indicated the presence of *Pseudomonas aeruginosa*, *Enterobacter cloacae*, *Achromobacter xylosoxidans*, and *Leclercia adecarboxylata.* Additionally, bioassays demonstrated the significant involvement of ACC deaminase. Among these microbes, *E. cloacae* significantly increased grain yield by up to 73% owing to the ACC deaminase activity (Table 5).

**TABLE 5:** Methanotrophs mitigate drought stress.

Country, year, references	Microbes	Host plant	Effect on drought stress	Mechanism of action	Isolation
India, 2019, (Kumar et al., 2019)	Methylotrophic bacteria (*Methylarcula, Methylobacillus, Methylobacterium, Methylocapsa, Methylocella, Methyloferula, Methylohalomonas, Methylomonas, Methylophilus, Methylopila, Methylosinus, Methylotenera*, and *Methylovirgula*)	*Glycine max*	Tolerant	↑auxin and cytokines ↑nitrogen fixation ↑ethylene ↑ACC deaminase ↑mineral solubilization	Phyllospheres of cotton and maize and earthworms in Tamil Nadu soil
Pakistan, 2020, (Danish et al., 2020)	*Pseudomonas aeruginosa, Enterobacter cloacae, Achromobacter xylosoxidans*, and *Leclercia adecarboxylata*	*Zea mays*	Tolerant	↑ACC deaminase	Maize rhizosphere

*The ↑ and ↓ represent increase and decrease the response of specific mechanism.*

Methanotrophs metabolize methane from carbon molecules and produce sulfate, nitrate, and oxidation energy. They require only a single carbon atom for survival; therefore, their survival rate is higher.

### Mycorrhizae

Mycorrhizae are microbial symbiotes that maintain a symbiotic association with plants. They are host-specific and have interspecific functionality (Sheteiwy et al., 2021). Arbuscular mycorrhizae facilitate host plant survival by increasing water and nutrient absorption from the rhizosphere (Agapitos et al., 2008), whereas endophytes do so by providing phytohormones and inducing defense-related secondary metabolism within the plants (Barman et al., 2016; Cheng et al., 2021; Kozjek et al., 2021).

In a study on soybean, which is highly sensitive to abiotic stress, plants were inoculated with arbuscular mycorrhizal fungi (AMF); the isolates were obtained from Argentina (Grümberg et al., 2015). After inoculation, the plants were subjected to drought conditions, and their physiological, biochemical, and molecular characteristics were evaluated. The quantitative analysis showed increased levels of proline, soluble sugars, and glycine betaine and reduced levels of MDA. MDA is a product of polyunsaturated fatty acids in cells and serves as the gold standard for identifying oxidative stress. The fungal strains *Septoglomus constrictum, Glomus* sp., and *Glomus aggregatum* effectively increase the levels of osmoprotectants in plant leaves and provide potential protection against drought stress (Grümberg et al., 2015).

Apart from abiotic stress, drought stress is the leading cause of plant diseases and limits crop production. In a randomized trial conducted by Cheng et al. (2021), *Funneliformis mosseae* was used to colonize trifoliate orange. After treatment for 8 weeks, a dramatic increase was observed in the leaf number, leaf area, stem elongation, and root microenvironment of the plants. Additionally, the qualitative analysis revealed increased phenolic, coumarin, and terpene contents in the root exudates. Phenolic components are key elements of drought stress tolerance, as they reduce oxidative stress (Cheng et al., 2021). In another study, two cultivars of wheat bread and durum wheat were selected and inoculated with *Glomus mosseae*, a strain of mycorrhizal fungi, and then subjected to water restriction (Bernardo et al., 2017). In a randomized controlled trial, analyses of growth parameters and proteomics were conducted to determine the mechanisms of drought tolerance. The results suggest that inoculation increased the accumulation of aboveground dry biomass. Furthermore, an increased genotype diversity is related to stress tolerance. Various genes modulate the signaling pathways of sulfur and oxylipin metabolism, which inhibits ethylene formation. Ethylene causes plant stress and damages cell integrity and osmotic pressure. Downregulation of 6-SFT expression was also noted. The upregulation of osmolytes in the cellular components of plants was significant, indicating that oxidation–reduction mechanism is associated with drought tolerance (Bernardo et al., 2017).

Microbes can be ecofriendly and aid in stress tolerance in plants. Drought stress involves a cascade of events that cause oxidative damage. Thus, the plant antioxidant defense system is activated by the induction of gene expression to combat ROS. For example, inoculation of two AMF strains of *Gigaspora margarita* and *Glomus intraradices* in host plants resulted in the expression of *GmarCuZnSOD, GintPDX1, GintMT1*, and *GintSOD*. In addition, the mechanism for tolerating drought stress involves the reduction of cytoplasmic protein levels and regulation of redox status *via* pyridoxamine synthesis (Zou et al., 2021).

Drought stress negatively affects the crop quality and quantity. However, this can be mitigated through the inoculation of three species of AMF, namely, *G. mosseae, Glomus etunicatum*, and *G. intraradices*. Al-Arjani et al. (2020) isolated these three species from the rhizosphere of *Acacia gerrardii* and applied them to *Ephedra foliata* Boiss plants. Consequently, plants were preserved under restricted water conditions (used to induce drought stress). Specifically, compared with control plants, the inoculated plants were more likely to show photoassimilation, which increased the chlorophyll and carotenoid contents. Additionally, increased levels of sucrose-phosphate synthase were observed in the carbon pool. Furthermore, increased osmolytes are among other factors attributed to drought stress tolerance (Al-Arjani et al., 2020). In another study, AMF and silicon were inoculated with 1-week-old strawberry seedlings to observe their synergistic effects against drought stress (Moradtalab et al., 2019). Following a randomized trial over 4 weeks, AMF and silicone were found to increase the biomass, water uptake, and mineral content and promote the antioxidant defense system. Thus, the two are beneficial for combating drought stress when combined (Moradtalab et al., 2019; Table 6).

**TABLE 6:** Mycorrhizae mitigate drought stress.

Country, year, references	Microbes	Host plant	Effect on drought stress	Mechanism of action	Isolation
Iran, 2021, (Moradtalab et al., 2019)	Arbuscular mycorrhizal fungus	*Fragaria × ananassa* Duch.	Mitigate	↑Zn ↑AA enzyme ↑water uptake	Pal Axel Lab, Lund University, Sweden
Argentina, 2014, (Grümberg et al., 2015)	Arbuscular mycorrhizal fungi	*Glycine max*	Tolerance	↑osmoprotectants ↑proline, glycine, and soluble sugars ↓MDA	Ecosystem, experimental field Central Argentina
China, 2021, (Cheng et al., 2021)	*Funneliformis mosseae*	*Trifoliate orange*	Stress modulation	↑phenolic contents ↑terpenes ↑root exudate ↑coumarins ↓alkanes, ester, and amides	Rhizosphere Jingzhou, China
Italy, 2017, (Bernardo et al., 2017)	Arbuscular mycorrhizal fungus (*Glomus mosseae*)	*Triticum* spp.	Tolerance	↓6-SFT ↓SOD ↑genetic diversity ↓sulfur metabolism	MycAgro Lab (Techno pole Agro-Environment, Bretenière, France)
Saudi Arabia, 2020, (Al-Arjani et al., 2020)	AMF (*Glomus etunicatum*, *Glomus intraradices*, and *Glomus mosseae*)	*Ephedra foliata* Boiss	Tolerance	↑gene expression ↑mineral solubilization ↑phytohormone production ↑transport protein expression ↑osmolytes ↑antioxidant enzyme	Rhizosphere of *Acacia gerrardii*

*The ↑ and ↓ represent increase and decrease the response of specific mechanism.*

Mycorrhiza form a symbiotic relationship between fungi and plants. The term mycorrhiza refers to the role of the fungus in the plant axis; mycorrhizae play a significant role in plant growth, nutrition, soil biochemistry, and stress tolerance.

## Growing Interest

**FIGURE 5:**
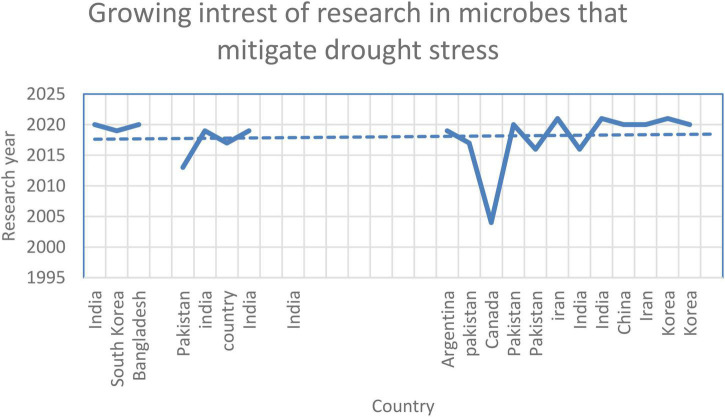
Growing interest in research related to microbes that mitigate drought stress.

There is an increasing trend in publications related to this topic, which will likely continue to increase owing to the need to identify adaptive solutions that can determine the effects of climate change on crops. Notably, several studies have used a mixture of diverse microbial strains, and it has been speculated that a consortium would be more archetypal of the original microbiome, which would include numerous strains that can provide distinctive synergistic benefits compared with individual strains (Figure-5).

## Conclusion and Future Perspectives

Plants have evolved in a sophisticated manner with microbes to overcome drought stress and its adverse effects within cells. Microbes strengthen the intrinsic stress tolerance mechanisms of plants by producing exopolysaccharides, phytohormones, ACC deaminase, osmolytes, and volatile compounds. In addition, cells synthesize diverse molecules that regulate redox damage and overcome antioxidant damage.

This review highlights a variety of adverse effects of drought stress on plant growth and reveals how such effects can be mitigated using microbes. Previous reviews have focused on drought mitigation by using a group of microbes. However, this review discusses a variety of microbes that mitigate drought stress, their fundamental mechanisms of action, and the changes that they produce at the molecular level. Microbial intervention has led to substantial progress in drought tolerance. Future investigations should evaluate whether acute and chronic effects are beneficial to plant phenotypes. Although known microbial interventions are promising, their dose, frequency, and timing warrant practical validation.

## Author Contributions

SS and MAK contributed to the original draft writing. MI and S-MK helped with review and graphical representation. Y-SP and SHW helped in formatting the manuscript. I-JL helped with funding. All authors contributed to the article and approved the submitted version.

## Conflict of Interest

The authors declare that the research was conducted in the absence of any commercial or financial relationships that could be construed as a potential conflict of interest.

## Publisher’s Note

All claims expressed in this article are solely those of the authors and do not necessarily represent those of their affiliated organizations, or those of the publisher, the editors and the reviewers. Any product that may be evaluated in this article, or claim that may be made by its manufacturer, is not guaranteed or endorsed by the publisher.

## Abbreviations

AMF: Arbuscular mycorrhizal fungi
MDA: Malondialdehyde
LB: Lysogeny broth
PGPR: Plant-growth-promoting rhizobacteria
PGPB: Plant-growth-promoting bacteria
RIDER: Rhizobacteria-induced drought endurance and resilience
SOD: Superoxide dismutase
ROS: Reactive oxygen species
TPP: Trehalose-6-phosphate phosphatases
ABA: Abscisic acid
IAA: Indole acetic acid
ACC: Aminocyclopropane-1-carboxylic acid
VOC: Volatile oil compounds.
